# Neuroimmune responses in the chicken brain during acute fowl adenovirus serotype 4 infection

**DOI:** 10.3389/fvets.2026.1964202

**Published:** 2026-09-23

**Authors:** Ulfiani Fauzia Hanafi, Rangyeon Lee, Kyungseok Shin, Yoon-Beom Lee, Geelsu Hwang, Lilik Maslachah, Jeongho Park

**Affiliations:** 1 College of Veterinary Medicine, Kangwon National University, Chuncheon, Gangwon-Do, Republic of Korea; 2 Multidimensional Genomics Research Center, Kangwon National University, Chuncheon, Gangwon-Do, Republic of Korea; 3 Department of Preventive and Restorative Sciences, School of Dental Medicine, University of Pennsylvania, Philadelphia, PA, United States; 4 Department of Veterinary Basic Medicine, Faculty of Veterinary Medicine, Universitas Airlangga, Surabaya, East Java, Indonesia

**Keywords:** brain immunity, FAdV-4, non-neurotropic virus, non-target organ infection, tight junction proteins

## Abstract

**Introduction:**

Fowl adenovirus serotype 4 (FAdV-4) causes fatal hepatitis in chickens and has a substantial impact on the poultry industry. The virus primarily targets the liver in chickens. Although viral load in the brain has been previously reported, the association of acute FAdV-4 infection with brain immune response remains poorly characterized. Therefore, this study focuses on immune alterations in the brain during acute FAdV-4 infection.

**Methods:**

Here, we investigated an increase in immune cell populations, along with the expression of barrier-related genes, to define the neuroimmune response during FAdV-4 infection.

**Results:**

Our results demonstrated that there was an alteration in the immune cell population, characterized by an increased frequencies of activated monocytes/macrophages and T cell subsets in the brain from infected chickens. Additionally, the CD44^+^CD8^+^ and γδ CD8^+^ were elevated among T cell subsets and accompanied by a surge in *TNFA* mRNA expression. The alteration was extended to the low expression of genes encoding tight-junction (TJ) proteins, such as *TJP1, CLDN5,* and *OCLN*, and a depletion in CLDN5 protein levels. This downregulation corresponded to the detection of FAdV-4 hexon DNA in the chicken brain homogenates from the infected group, suggesting a potential link between structural barrier alterations and viral presence.

**Discussion:**

Together, these findings suggest acute FAdV-4 infection-associated neuroimmune alterations, mapping a distinct immunophenotypic and transcriptional landscape within the avian brain.

## 1 Introduction

The Adenoviridae family comprises non-enveloped, icosahedral viruses with linear double-stranded DNA genomes that infect a wide range of vertebrate hosts, including mammals, birds, reptiles, amphibians, and fish (1, 2). The viral capsid is primarily composed of hexon and fiber proteins (3), which are key determinants of viral infectivity and tissue tropism. Fowl adenovirus serotype-4 (FAdV-4) is of particular concern because its outbreaks cause severe disease and substantial economic losses in the poultry industry (4).

FAdV-4 primarily targets the liver, where its replication leads to acute and often fatal hepatitis (5–7). This virus transmits rapidly among hosts via the fecal–oral route, with higher viral loads detected in cloacal swabs than in oropharyngeal samples (6, 8). Following initial infection, the virus rapidly disseminates through the bloodstream, leading to systemic infection within the host tissues. Circulating monocytes/macrophages increase markedly at 3 days post-infection (dpi), while CD8^+^ T cells peak at 5 dpi (9). Besides, hepatic T lymphocytes rise significantly by 3 dpi (7), suggesting that FAdV-4 infection promotes activation and migration of macrophages and T cells locally and systemically.

While the liver is the primary site of infection, FAdV-4 has been detected in other organs, including the brain, as confirmed by the presence of viral DNA in brain tissue (10). Although viral load and histopathological lesions, such as encephalitis and perivascular lymphocytic infiltration, are present in the brain, FAdV-4 does not alter CARD11 levels, a biomarker typically associated with neurotropic viral invasion (10–12). This suggests that while FAdV-4 is present in the CNS, its pathogenicity may differ from classical neurotropic viruses. In general, viral neuroinvasion can increase BBB permeability by downregulating tight junction (TJ)-associated proteins such as *OCLN*, *CLDN5*, and zonula occluden-1 (*ZO1*) (13, 14). This is often accompanied by microglial activation and the infiltration of peripheral immune cells into the brain (15, 16). Whether such barrier and immune alterations occur during infection of FAdV-4 remains to be fully elucidated. Therefore, the objective of this study is to investigate how FAdV-4 infection affects immune homeostasis within a non-primary target organ, specifically focusing on the avian brain.

In this study, we examined population of immune cells and TJ-related mRNA and protein level in the brain during acute FAdV-4 infection. Our results demonstrated a distinct expansion of macrophages and T cell subsets, such as a higher frequency of MHCII-expressing macrophages and the accumulation of cytotoxic T cell populations, including CD44^+^CD8^+^ and γδ CD8^+^. Besides that, a significant reduction in the expression of TJ-associated genes and proteins, such as CLDN5, was observed. This downregulation also coincided with the detection of FAdV-4 DNA in the brain homogenate samples, suggesting that brain undergo distinct neuroimmune and transcriptional alterations during viral dissemination.

## 2 Materials and methods

### 2.1 Virus preparation

The FAdV-4 virus strain was isolated and propagated as described in previously published papers (7, 17). The FAdV-4 virus was isolated from 45-week-old FAdV-4-infected layers in Gyeonggi-do, Republic of Korea. The virus was subsequently propagated through 10 serial passages in Leghorn male hepatocellular carcinoma (LMH) cells, a chicken hepatocellular carcinoma cell line obtained from ATCC (CRL-2117). The cells were maintained in Waymouth’s growth medium (Gibco, United States), supplemented with 1% antibiotic-antimycotic solution (Corning, United States) and 10% fetal bovine serum (FBS; Corning, United States).

### 2.2 Animals and virus inoculation

Forty-four SPF chickens, aged 1 day, were purchased from Namduck SPF (Seongnam, Gyeonggi, Republic of Korea) and randomly divided into two groups: control (*n* = 14; 4–5 per experiment) and FAdV-4 or infection group (*n* = 31; 9–12 per experiment). They were placed separately in isolation cages. Their diet and drink were provided ad libitum. By day 8, the infection group was inoculated intramuscularly. The virus stock had a titer of 10^5^ TCID_50_/mL. The virus stock was diluted with PBS at a virus-to-PBS ratio of 1.5:8.5, and 100 μL of the diluted virus suspension was inoculated into each chicken. Due to the high pathogenicity of the virus, significant mortality was observed. Dead chickens (*n* = 21) were excluded from sampling to avoid post-mortem artifacts. At 5 dpi, the remaining chickens in the infected group (*n* = 7; 2–3 per experiment) and the control group (*n* = 10; 3–4 per experiment) were sacrificed by cervical dislocation. The experiment was triplicate. The experimental design and sampling timeline are summarized in Figure 1A.

**Figure 1:**
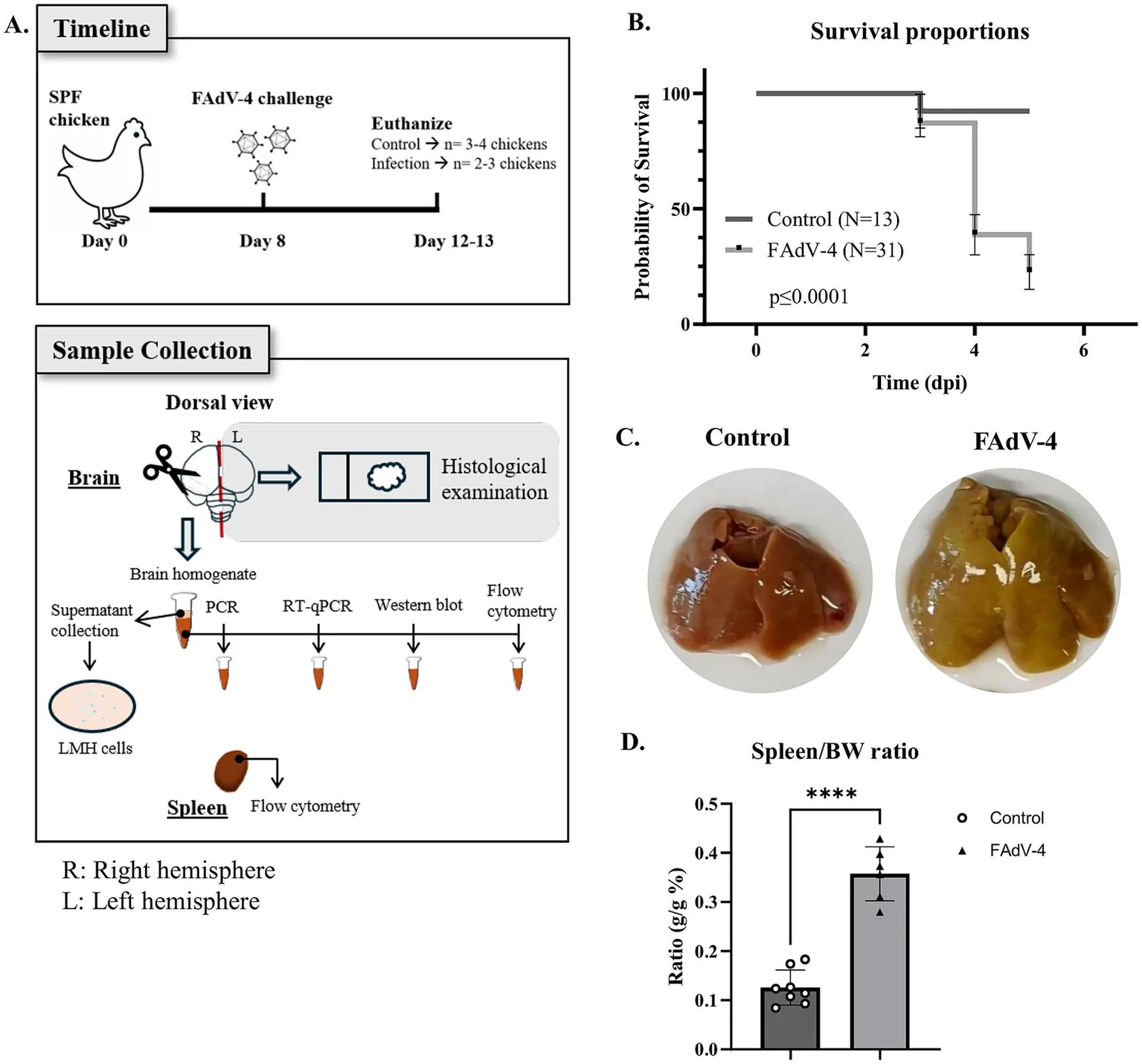
Experiment workflow and clinical outcomes following FAdV-4 infection. **(A)** Schematic representation of the experimental design and sample collection timeline. Following the infection, the infected group showed **(B)** a low survival rate, **(C)** severe hepatitis, and **(D)** a higher spleen-to-body weight ratio. **** *p* < 0.0001.

The tissue samples were collected for further examination as illustrated in Figure 1. Each brain was divided along the mid-sagittal plane into two hemispheres. The right hemisphere was immediately processed for immune cell isolation, DNA, RNA, and protein extraction, while the left hemisphere was prepared for histological analysis. All animal procedures were conducted in accordance with the guidelines of the Institutional Animal Care and Use Committee of Kangwon National University, and the study protocol was approved under Approval No. KW-210401-1, KW-241007-1.

### 2.3 Immune cell preparation

Brain homogenate tissues were dissociated with collagenase type IV (Worthington Biochemical Corporation, United States) for 45 min at 37 °C in an incubator. The dissociated samples were passed through a 40-μm nylon cell strainer (SPL Life Sciences, 93,040) and then centrifuged. The pellets were resuspended with a 30% Percoll (Sigma-Aldrich, United States) gradient solution. The gradient density dissociation was performed by slowly adding a suspension of sample pellets to a 30% Percoll gradient, coating the top of the 70% Percoll gradient solution. Percoll gradient centrifugation performed with no brake and no acceleration at 25 °C for 25 min, 2000 rpm. Red blood cells were lysed with RBC lysis buffer for 2 min, then washed with PBS (Gibco, United States). Isolated cells from each sample were centrifuged and resuspended in RPMI complete media. Subsequently, cell counts were performed with a hemocytometer.

Immune cell preparation from spleen tissue was performed using the same methods, except for the collagenase and Percoll gradient steps. The whole spleen tissue was used in this process. RPMI complete media used in this experiment was made from RPMI 1640 (L-glutamine and HEPES included) (Corning, United States) supplemented with 10% FBS (Corning, United States), 1% antibiotic-antimycotic solution (Corning, United States), 1 mM sodium pyruvate (Gibco, United States), 1% non-essential amino acid (Gibco, United States), and 0.1% β-Mercaptoethanol (Gibco, United States).

### 2.4 DNA extraction and viral detection in conventional PCR

100 μL of brain homogenate was centrifuged, the pellet was resuspended in 100 μL of PBS, and kept at −70 °C until later use. After thawing, the DNA from homogenized tissue was extracted using the DNeasy Blood and Tissue Kit (Qiagen, Germany) according to the manufacturer’s protocol. The concentration of isolated DNA samples was measured using a NanoDrop spectrophotometer (Thermo Fisher, United States).

For virus detection by conventional PCR, AccuPower PCR Premix (Bioneer, South Korea) was used with 20 μL total PCR mixture containing 1,000 ng of extracted DNA and 2 μL of FAdV-4 *hexon* primers. *Hexon* primer sequences used in this study are listed in Table 1. The steps refer to the manufacturer’s protocol. The initial denaturation was set at 95 °C for 5 min. Cyclic conditions consisted of 35 cycles at 95 °C for 20 s, 58 °C for 20 s, and 72 °C for 1 min, followed by final extension at 72 °C for 5 min. The PCR products were separated by electrophoresis on a 2% agarose gel (Bioneer, South Korea) prepared in 1X TAE running buffer (Bioneer, South Korea). The bands were visualized with ethidium bromide.

**Table 1:** List of primers’ sequences used in this study.

Gene	Sequences	Length (bp)	Accession number
FAdV-4 Hexon	F: CAARTTCAGRCAGACGGT R: GAAATCTTCGTCACCTGCG	384	HQ709228.1
*ACTB*	F: TTGTCCACCGCAAATGCTTC R: AAGCCATGCCAATCTCGTCT	107	NM_205518.2
*TNFA*	F: AGATGGGAAGGGAATGAACC R: ACTGGGCGGTCATAGAACAG	120	NM_204267.1
*IL6*	F: CCAGAAATCCCTCCTCGCCAATC R: GCCCTCACGGTCTTCTCCATAAAC	111	NM_204628.1
*IFNA*	F: ATCCTGGACACCAGCAACACCC R: CATCCTGCTGCTCACGCTCCTTCTG	198	EU334503.1
*IFNB*	F: CCTCCAGCTCCTTCAGAATACG R: ACAGCCTCCTCAACCAGATCCAGC	224	AY974089.1
*CXCL13*	F: CTGCCGGAGGAAAGAGATCA R: ACATCCGTCTGTGTCAGCTT	111	NM_001348657.2
*CCL5*	F: ACCGTGTGCTGCTTCAACTA R: TGAACACAACTGCTGCCTGT	103	NM_001045832.2
*CCR5*	F: GTGGTCAACTGCAAAAAGCA R: GCACGTTCAACTGTGTCG	190	NM_001045834.1
*TMEM119*	F: CATCGCTTGTGCTCTCTCCA R: CATCTGCGAGAAAGCTGTGC	109	XM_040684687.2
*P2RY12*	F: TGGGTGCTGAGAGGATTTGT R: AACGGTGAGGTGGCTTTCT	122	XM_040679503.2
*CX3CR1*	F: TCCAGAACGATCAAGCACAG R: CGGTGTTCAGTTCCACATTG	175	XM_418820.2
*TJP1 (ZO1)*	F: TATGAAGATCGTGCGCCTCC R: TGCCGGATAATAGCTGCGTT	159	XM_015278977.3
*CLDN5*	F: TGTCAGCCTTCATCGACGT R: TGGAATCGTACACCTTGCAC	114	NM_204201.2
*OCLN*	F: TCCTCATCGTCATCCTGCTC R: TTCTTCACCCACTCCTCCAC	145	NM_205128.1
*MMP-9*	F: ACCGTGCCGTGATAGATGAT R: AATCTGGGTGAAGGTGAGGG	80	NM_204667
*CARD11*	F: GCTTCTGACACGGCAGCATCT R: CATTTCCTTTAGCCCAATCCTC	195	XM_046927348.1

### 2.5 Histological examination

Left hemisphere of the brain was fixed in optimal cutting temperature (OCT) compound (Sakura Finetek, United States) immediately after collection and stored at −70 °C until later use. The samples were cryosectioned into 10 μm slices and were stained with Diff-Quick solution (Thermo Fisher, United States). The sectioned samples were observed at x40 magnification using a research inverted microscope (Eclipse Ts2R, Nikon, Japan), and images were captured using NIS Elements software. All images were quantified using ImageJ software for Windows.

For the immunofluorescent assay, 6–12 μm-thick tissue sections were briefly washed twice with PBS, and the tissue area was marked with a hydrophobic marker. Then it was fixed with 4% paraformaldehyde (PFA) for 10 min, washed in PBS twice, and then proceeded to the next step. 10% chicken serum in PBS was used for blocking non-specific binding, incubated at room temperature for 1 h. Following incubation, the sectioned samples were washed twice in PBS and stained with fluorescent antibodies for 1 h at room temperature. The mouse anti-chicken antibodies used in this examination included Monocyte/Macrophage-PE (KUL01) and CD8α-FITC (CT-8). All stained samples were washed in PBS twice after the incubation, and sections were mounted using Vectashield plus DAPI (Vector Laboratories, United States). The stained tissue slides were observed at x20 magnification using a confocal microscope (Leica Microsystems, Germany), and the images were visualized using Leica Application Suite X (LAS X) software. The obtained data were then analyzed using ImageJ software for Windows.

### 2.6 Flow cytometry

Brain and spleen immune cells were stained with fluorescent-labeled antibodies for 30 min at 4 °C. The antibodies used were LIVE/DEAD Fixable Aqua Dead Cell Stain kit (Thermo Fisher, United States) and mouse anti-chicken antibodies: TCRγδ-AF488 (TCR-1), CD44-PE (AV6), CD4-PE/CY7 (CT-4), CD8α-APC (CT-8), MHC class II-FITC (2G11), CD45-APC (LT40), Bu-1-PACBLU (AV20), CD3-AF700 (CT-3), and Monocyte/Macrophage-PE (KUL01) from Southern Biotech, United States. Magnetic-activated cell sorting (MACS) buffer was used for the staining and washing step, made by mixing PBS, 2% FBS, and 2 mM EDTA. Stained cells were analyzed using a CytoFLEX flow cytometry (Beckman Coulter, United States), and the data were analyzed with FlowJo v10.10.0 (Becton Dickinson, United States).

### 2.7 RNA extraction and real-time quantitative PCR

The brain homogenate was centrifuged to remove the medium, then 100 μL of PBS was added, followed by 400 μL of TRIzol reagent (Invitrogen, United States). The homogenate samples were then stored at −70 °C. For RNA extraction, TRIzol-homogenate brain mixture samples were thawed at room temperature, and an additional 400 μL of TRIzol reagent and 200 μL chloroform (Merck, Germany) were added after thawing, and the samples were vortexed thoroughly before centrifugation at 12,000 rcf for 15 min at 4 °C. The aqueous phase was recovered, mixed with 500 μL of isopropanol (Sigma-Aldrich, Germany), inverted 3–4 times, and then incubated for 10 min at RT. RNA was pelleted by centrifugation (12,000 rcf, 15 min, 4 °C), washed with 75% ethanol (Sigma-Aldrich, United States) followed by centrifugation at 7,500 rcf for 5 min, 4 °C, air-dried, and resuspended in 100 μL of nuclease-free water (Invitrogen, United States). The concentration of the isolated RNA was determined using a NanoDrop spectrophotometer (Thermo Fisher, United States).

A total of 1,000 ng of RNA was reverse-transcribed into cDNA using Maxima Reverse Transcriptase (Thermo Fisher Scientific, United States) with oligo(dT)20 primers (Invitrogen, United States). Following reverse transcription, cDNA was diluted with nuclease-free water and used as the template for RT-qPCR. RT-qPCR was performed in a 20 μL reaction mixture using Maxima SYBR Green/ROX qPCR Master Mix (Thermo Fisher, United States). All reactions were performed in duplicate. Threshold cycle (Ct) values were obtained using the QuantStudio™ 3 Real-Time PCR system (Thermo Fisher, United States). The expression level was calculated using the formula 2^−∆∆CT^. The mRNA level of *ACTB* was used as a housekeeping gene. The primer sequences used in this study are listed in Table 1. The sequence of primers was obtained from previously published papers (7, 18) or designed using Primer3 and BLAST (NCBI), and then synthesized based on the genome sequences in NCBI.

### 2.8 Protein extraction and western blotting

The total proteins of the brain-homogenate sample were extracted. Samples were mixed with radio immunoprecipitation assay (RIPA) buffer (LPS solution, South Korea) and incubated on ice for 5 min. Then, the samples were sonicated to facilitate cell lysis and protein extraction. Centrifugation at 13,000 rpm at 4 °C for 6 min was performed, and the supernatant was transferred to a new tube. Protein concentration was analyzed using the BCA Protein Assay kit (Thermo Fisher, United States).

Proteins were separated by 10% (for ACTB) or 15% (for CLDN5) sodium dodecyl sulfate-polyacrylamide gel electrophoresis (SDS-PAGE) and subsequently transferred onto a polyvinylidene fluoride (PVDF) membrane (Millipore, United States). The blocking step was performed by incubating the membranes with the blocking solution, EzBlock Chemi (ATTO, Japan), for 20–30 min while shaking at 80 rpm. After blocking, the membranes were washed 3 times with 1X tris-buffered saline with Tween-20 (TBST) for 5 min each and incubated with primary antibodies overnight at 4 °C at 80 rpm. Primary polyclonal antibodies against CLDN5 (Invitrogen, United States, 1:1000) and β-ACTIN (Bioss, China, 1:5000) were used. The membranes were then incubated with HRP-conjugated Goat anti-rabbit IgG (H + L) (Abclonal, China) for 1–3 h at RT, shaking at 120 rpm. Protein bands were detected by enhanced chemiluminescence (ECL) (Millipore, United States), imaged with the Gel documentation system, and quantified using ImageJ.

### 2.9 Viral isolation in LMH cells

A tissue homogenate from positive samples was centrifuged at 4 °C and 1,000 rpm for 4 min. The supernatant was transferred and pooled into a new tube. The tube was then stored at −70 °C. LMH cells (2×10^4^ cells/well) were seeded in 96-well plates as described in a previously published paper (19). The cells were incubated at 37 °C in 5% (v/v) CO_2_ for 1 or 2 days. Next, the LMH cells were inoculated with 30 μL of supernatant per well, and virus adsorption was done by incubating for 1 h at 37 °C in 5% (v/v) CO_2_. After incubation, the supernatant was removed and resuspended with 100 μL/well Waymouth’s medium. Cytopathic effects were observed using Incucyte^®^ (Sartorius, Germany) at 37 °C in 5% (v/v) CO_2_.

### 2.10 Statistics

Statistical significance was analyzed by GraphPad Prism software for Windows version 10. The log-rank test was used to analyze the survival rate and Fisher’s exact test was used for the analysis of percentage viral detection. Data were tested for normality using the Shapiro–Wilk test. For comparisons between two groups, an unpaired t-test was used when data were normally distributed and variances were equal. Welch’s *t*-test was applied for data that have unequal variances. The Mann–Whitney U test was used for data that was not normally distributed. For relative Ct values, the mean Ct value of the duplicated was used for further analysis to represent each individual biological sample. Statistical significance was determined using Two-Way ANOVA. Significant differences are indicated on the graph, with *p*-values throughout this article: * *p* < 0.05, ** *p* < 0.01, *** *p* < 0.001, and **** *p* < 0.001.

## 3 Results

### 3.1 Clinical symptoms and the change in the organs during acute fowl adenovirus serotype-4 infection

To evaluate the impact of acute FAdV-4 infection on brain, chickens were inoculated according to the experimental design outlined in Figure 1A. Following challenge, the chickens exhibited clinical signs such as lethargy, anorexia, and weight loss beginning at 3 days post-infection (dpi). Mortality was initiated at 3dpi and reached its highest rate at 4 dpi (Figure 1B). We also observed yellowish liver tissue following the viral challenge at 5 dpi, indicating liver injury with the typical lesions of FAdV-4 infection in chickens. In contrast, the non-infected group did not exhibit gross pathological lesions (Figure 1C).

Following viral dissemination into multiple organs, FAdV-4 is known to affect the lymphoid organs, causing spleen oedema and congestion during FAdV-4 infection (11). We detected the presence of FAdV-4 *hexon* DNA in the spleen sample of infected chickens (Supplementary Figures S1A). Next, we observed the spleen-to-body weight ratio to assess lymphoid tissue pathology. A significant increase in this ratio was observed at 5 dpi (Figure 1D), showing splenomegaly secondary to the viral challenge. To investigate further the consequences of FAdV-4 infection on systemic lymphoid tissues, we analyzed immune cell distributions within the spleen. Our results showed that both the frequency and absolute number of splenic immune cells, including activated monocytes/macrophages and T cells, were decreased compared with uninfected controls (Supplementary Figures S1B, S2A–C). These findings demonstrate that FAdV-4 pathogenesis extends to secondary lymphoid organ such as the spleen, beyond its primary target organ, the liver.

### 3.2 Brain immune cells expansion observed in histological examination

Having observed FAdV-4 pathogenesis in multiple organs, including the liver and spleen, we next examined a non-primary target organ, such as the brain. Since histopathological studies have previously documented brain lesions, such as vascular congestion and perivascular edema, within FAdV-4 infected chickens (11, 20), we examined the corresponding leukocyte dynamics in the brain from this group. Based on previous tissue-level alterations, particularly the accumulation of blood components within the cerebral vasculature, we hypothesized that the viral challenge might prompt a redistribution or affect the abundance of infiltrating subsets, including total leukocytes, lymphocytes, and myeloid cells. First, although significant differences in the number were not observed, monocyte/macrophages appeared enlarged in our histological examination (Figure 2A).

**Figure 2:**
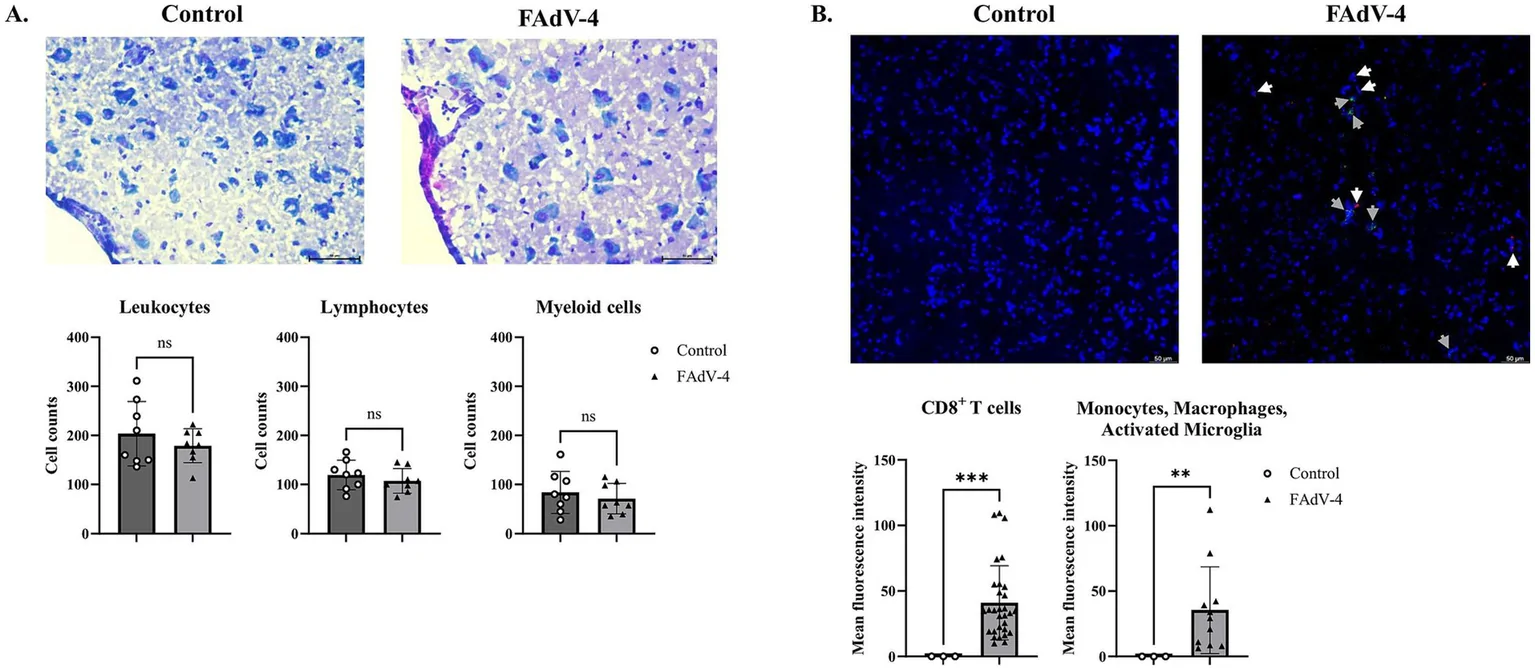
Infiltration of immune cells in the brain during FAdV-4 infection. **(A)** Diff-Quick-stained brain sections are shown at x40 magnification (scale bar = 50 μm). The graph shows the cell counts of leukocytes, lymphocytes, and myeloid cells. **(B)** Immunofluorescence assay shows monocytes/macrophages, marked with a white arrow, while CD8^+^ T cells are indicated with grey arrows. DAPI, stained with blue, showing a nuclear cell. Representative images were observed at x20 magnification, and the scale bar represents 50 μm (*n* = 2–3 individual experiments).

To further identify these increased immune cells, immunofluorescence (IF) staining was performed on brain sections specifically targeting macrophages and CD8^+^ T cells. As critical antigen-presenting cells that bridge innate and adaptive immunity, activated macrophages process viral pathogens for presentation via major histocompatibility complex (MHC) to activate T cells, such as cytotoxic CD8^+^ T cells (21). The results revealed a close contact of macrophages and lymphocytes in the forebrain. Stronger fluorescence signals corresponding to macrophages (red) and cytotoxic T cells (green) were detected in the infected group, predominantly around the edge of the forebrain (Figure 2B). Based on these IF examinations, we observed an accumulation of brain immune cells, particularly macrophages and CD8^+^ T cells, during FAdV-4 infection.

### 3.3 Increased brain macrophages during FAdV-4 infection

To precisely map and quantify the localized leukocyte dynamics, flow cytometry was utilized. The expression of major histocompatibility complex (MHC) class II was evaluated to monitor the activation state of the intracerebral monocyte/macrophage population. Circulating monocytes migrate to sites of tissue inflammation or damage to differentiate into macrophages, which serve as primary phagocytes that recognize pathogen-associated molecular patterns (PAMPs) and process antigens for MHC-mediated presentation (21, 22). At 4 dpi, the infected chickens showed an increase in MHC II–expressing macrophages in the brain; however, this change was not statistically significant in either frequency or absolute number (Supplementary Figure S3). By 5 dpi, however, the frequency of these cells increased significantly, while the absolute number showed a similar upward trend but did not reach statistical significance (Figure 3A).

**Figure 3:**
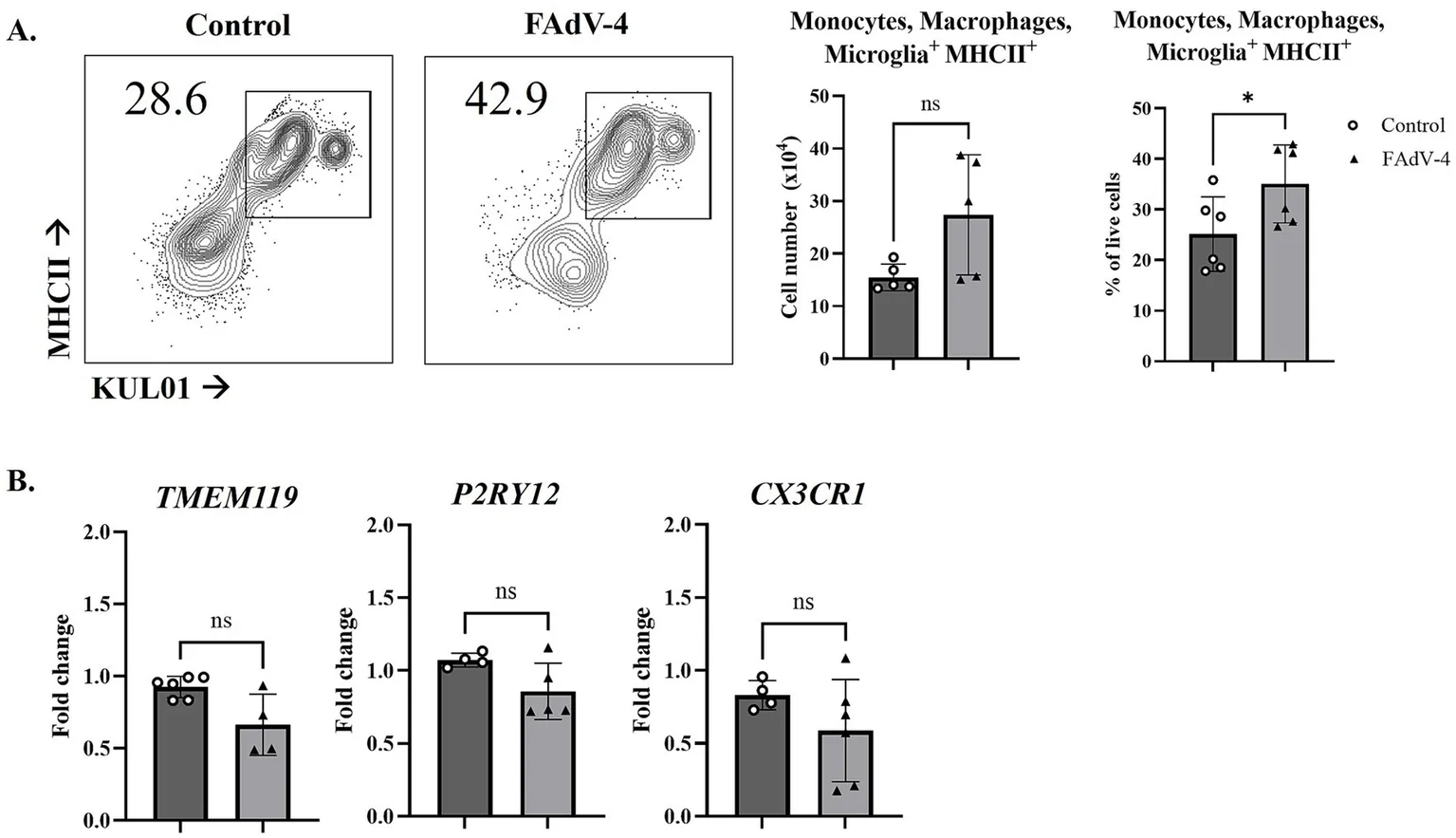
Changes in the population of activated macrophages during FAdV-4 infection. **(A)** The frequency and cell number changes of MHC II-expressing monocytes/macrophages/activated microglia were analyzed by flow cytometry. Cells were obtained from the brain homogenate tissue and collected from 2 to 4 chickens per group at 5 dpi. The mRNA expression of **(B)** microglia’s homeostatic-related genes at 5 dpi were assessed by RT-qPCR. The gene expression was normalized to *ACTB* gene and the fold changes were calculated. The experiment was performed in three independent experiments, and the pooled data are shown. * *p* < 0.05.

To further evaluate whether this macrophage expansion was from tissue-resident microglia or peripheral monocyte recruitment, the transcriptional levels of homeostatic microglia-specific markers were evaluated by RT-qPCR analysis. Upon microglial activation, *TMEM119*, *P2RY12*, and *CX3CR1*, which are markers for homeostatic microglia, are typically reduced (23). Although the mRNA expression levels of *TMEM119, P2RY12,* and *CX3CR1* did not display a statistically significant decrease at 5 dpi, a decreasing trend was observed (Figure 3B). Together with the significantly higher frequency of MHCII-expressing cells (Figure 3A), these data indicate an altered brain myeloid cells to the activation state during the acute FAdV-4 challenge.

### 3.4 Regulation of brain T cells during FAdV-4 infection

Given the altered activation profiles observed in the myeloid compartment, we next evaluated whether the T cells in the brain were also altered following the FAdV-4 challenge. Activated macrophages critically coordinate downstream adaptive immunity by influencing T cell recruitment and activation (22). We analyzed T cell subsets (CD3^+^) based on CD8 expression in combination with the activation marker CD44. In antiviral host defense, CD8^+^ T cells can release cytotoxic granules, such as perforin and granzymes, alongside proinflammatory cytokines to clear infected target cells (24). In the brains of the infected group, activated CD8^+^ T cells were not significantly increased at 4 dpi, whereas one day later, they were significantly elevated in both frequency and absolute number (Supplementary Figure S4A; Figure 4A). A previous study reported that chicken γδ CD8^+^ T cells exhibit cytotoxic activity rapidly, as these cells recognize antigens in an MHC-independent manner (25). We observed a slight, non-significant upward trend in CD44^+^ γδ T and CD8^+^ γδ T cells at 4 dpi (Supplementary Figures S4B,C). Their elevation became more pronounced at 5 dpi. CD44^+^ γδ T cells exhibited a significant increase in frequency, although their absolute numbers did not reach statistical significance (Figure 4B). Meanwhile, CD8^+^ γδ T showed a significant increase in both frequency and absolute number (Figure 4C). These findings indicate that FAdV-4 infection induces activation and expansion of cytotoxic T cell populations in the chicken brain, predominantly involving both CD8^+^ αβ T and CD8^+^ γδ T cells.

**Figure 4:**
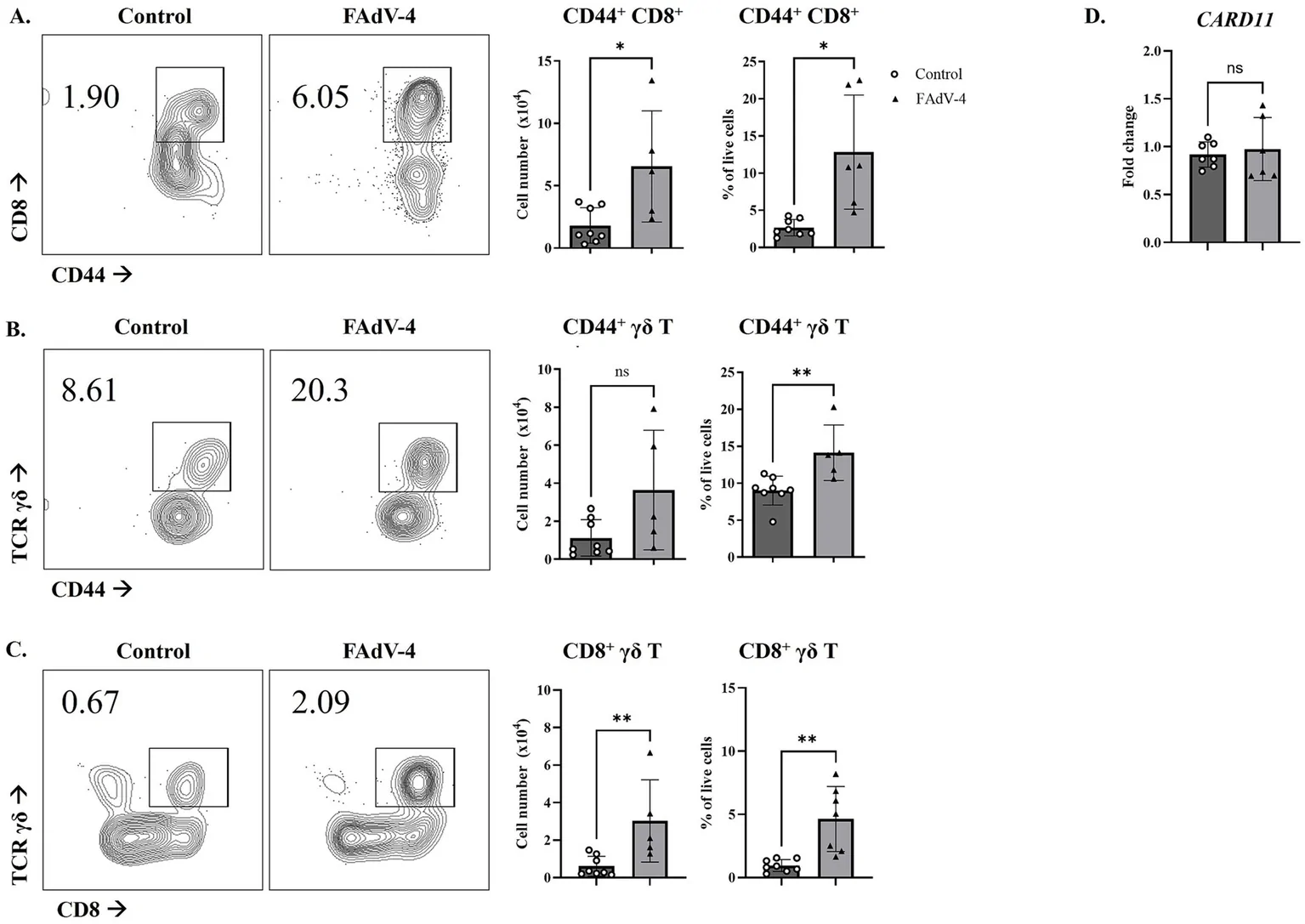
The alteration of activated T cells in the brain following FAdV-4 infection. The cell number and frequencies of **(A)** CD44^+^CD8^+^, **(B)** CD44^+^γδ T cells, and **(C)** γδ CD8^+^ T cells were presented. Representative plots from three independent experiments are shown. Cells were collected from 2 to 4 chickens per group at 5 dpi. **(D)** Fold change in mRNA expression of *CARD11* was assessed by RT-qPCR and normalized to the *ACTB* gene. * *p* < 0.05, ** *p* < 0.01.

To further investigate the activation state of these lymphocytes, we assessed the mRNA expression of caspase recruitment domain-containing protein 11 (*CARD11*). CARD11 initiates antigen receptor-mediated activation of the NF-κB pathway, thus regulating lymphocyte activation (26). By 5 dpi, *CARD11* expression was not significantly changed (Figure 4D), even as the absolute numbers of activated cytotoxic T cells and γδ T cells were increased, suggesting a temporary trigger signal in the spread of non-neurotropic virus into the brain tissue.

### 3.5 FAdV-4 infection induces pro-inflammatory gene expression in the brain

Next, the transcriptional levels in the infected group were examined and compared with those in the control group to evaluate host immune defense in the brain, including pro-inflammatory cytokine secretion, immune trafficking, and antiviral response. During viral challenges, tissue-resident and infiltrating macrophages initiate the innate immune cascade through the rapid secretion of type I interferons (IFNs) and downstream pro-inflammatory cytokines, thereby mediating immune cell recruitment into the site of inflammation (21). TNFA is an early pro-inflammatory cytokine, while IL6 is induced during later phases of inflammation (27). We observed a significant increase in *TNFA* mRNA expression, whereas the mRNA level of *IL6* remained unchanged in the brain of the infected group. Type I interferons, such as IFNB, mediate antiviral immunity (28). The gene expression of *IFNB* was reduced in the brains from infected animals compared with uninfected controls (Figure 5A), indicating the induction of an inflammatory response alongside the suppression of local antiviral immunity in the brain.

**Figure 5:**
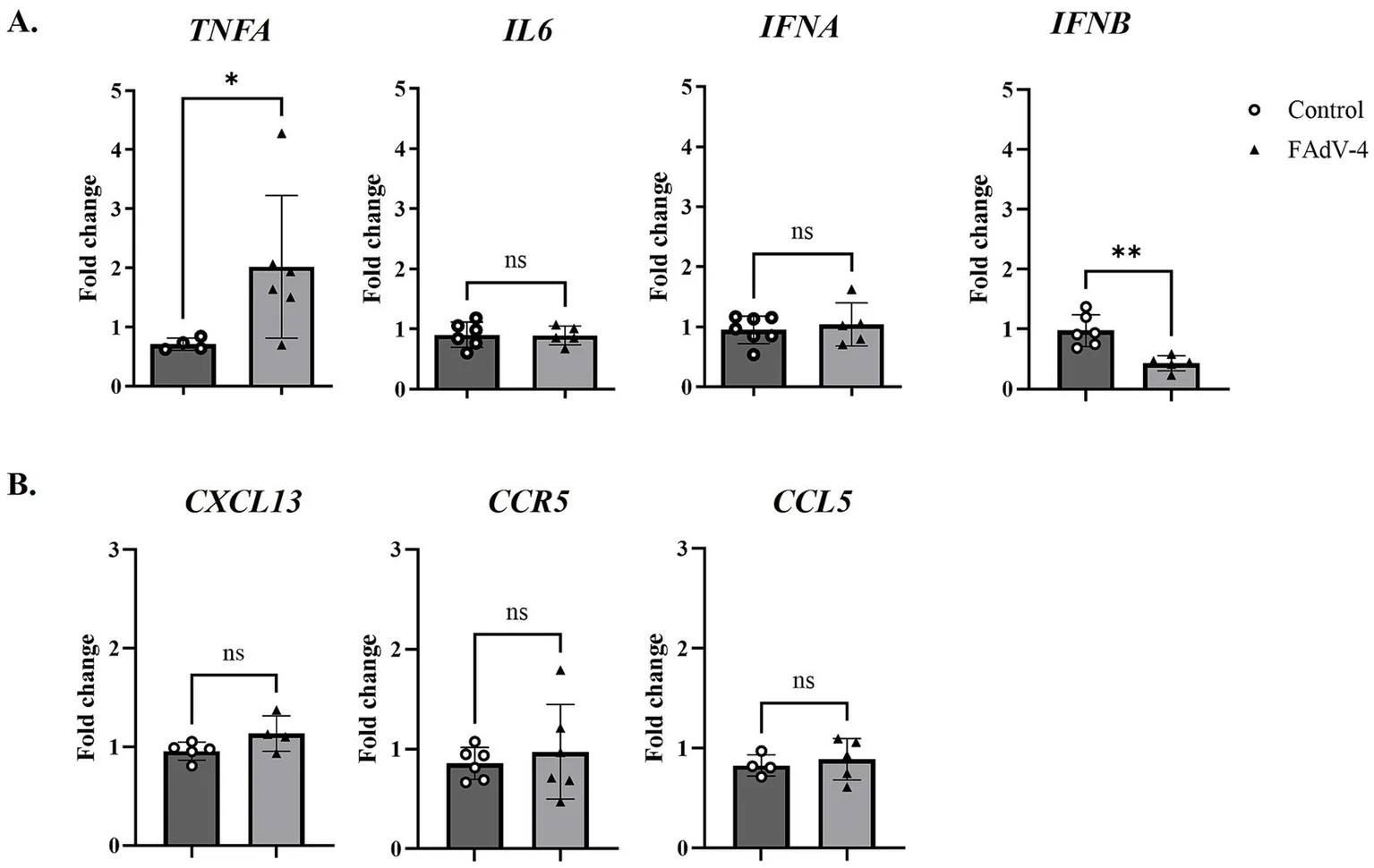
The mRNA level of cytokines and chemokines in the brain following FAdV-4 infection. The mRNA expression of **(A)** immune cell-associated cytokines and **(B)** chemokines at 5 dpi were assessed by RT-qPCR. Fold changes are presented with gene expression normalized to *ACTB* gene. The cDNA samples were obtained from homogenized tissue, and RT-qPCR was conducted in duplicate. Combined data from three independent experiments (*n* = 2–4 per group) are presented. * *p* < 0.05, ** *p* < 0.01.

Our analysis was further extended to chemokines involved in lymphocyte recruitment to sites of inflammation, such as *CCR5, CCL5*, and *CXCL13*. The chemokine CCL5 acts as a homing signal, guiding CCR5-expressing cells (including T cells and macrophages) to the site of inflammation and tissue repair (16, 29). Meanwhile, CXCL13 is a potent chemokine that actively recruits circulating B cells to the brain during neuroinflammatory conditions (30). The mRNA *CCR5, CCL5,* and *CXCL13* expression remained statistically unchanged (Figure 5B). These results showed that the signaling environment for peripheral leukocyte homing during FAdV-4 was minimal.

### 3.6 Alteration in tight junction-related genes in the brain during FAdV-4 infection

Inflammatory responses in mucosal and neural tissues are frequently accompanied by compromised barrier integrity, typically characterized by the downregulation of TJ proteins and increased matrix metalloproteinase (MMP) activity (16, 31, 32). Given our previous observation of *TNFA* mRNA upregulation, *IFNB* suppression, and increased macrophage activation in the brain of the infected group, we hypothesized that this inflammatory milieu might correlate with brain barrier alteration during FAdV-4 infection. This hypothesis is further supported by literature indicating a stage-dependent association between microglial/macrophage activation and CLDN5 expression (16).

To test this, we evaluated the expression of key TJ genes critical for maintaining brain barrier homeostasis, specifically *TJP (ZO-1)*, *CLDN5,* and *OCLN*. At 5 dpi, mRNA levels of *TJP1 (ZO1)*, *CLDN5*, and *OCLN* were significantly lower in the brains of infected chickens, but *MMP9* levels were unchanged (Figure 6A). We validated TJ expression at protein level. CLDN5, in the brain of infected animals was significantly reduced at 4 dpi (Figure 6B). These findings show that besides initiating inflammation response in the brain, FAdV-4 infection also downregulated TJ-related genes (TJP, CLDN5, and OCLN) and CLDN5 protein expression.

**Figure 6:**
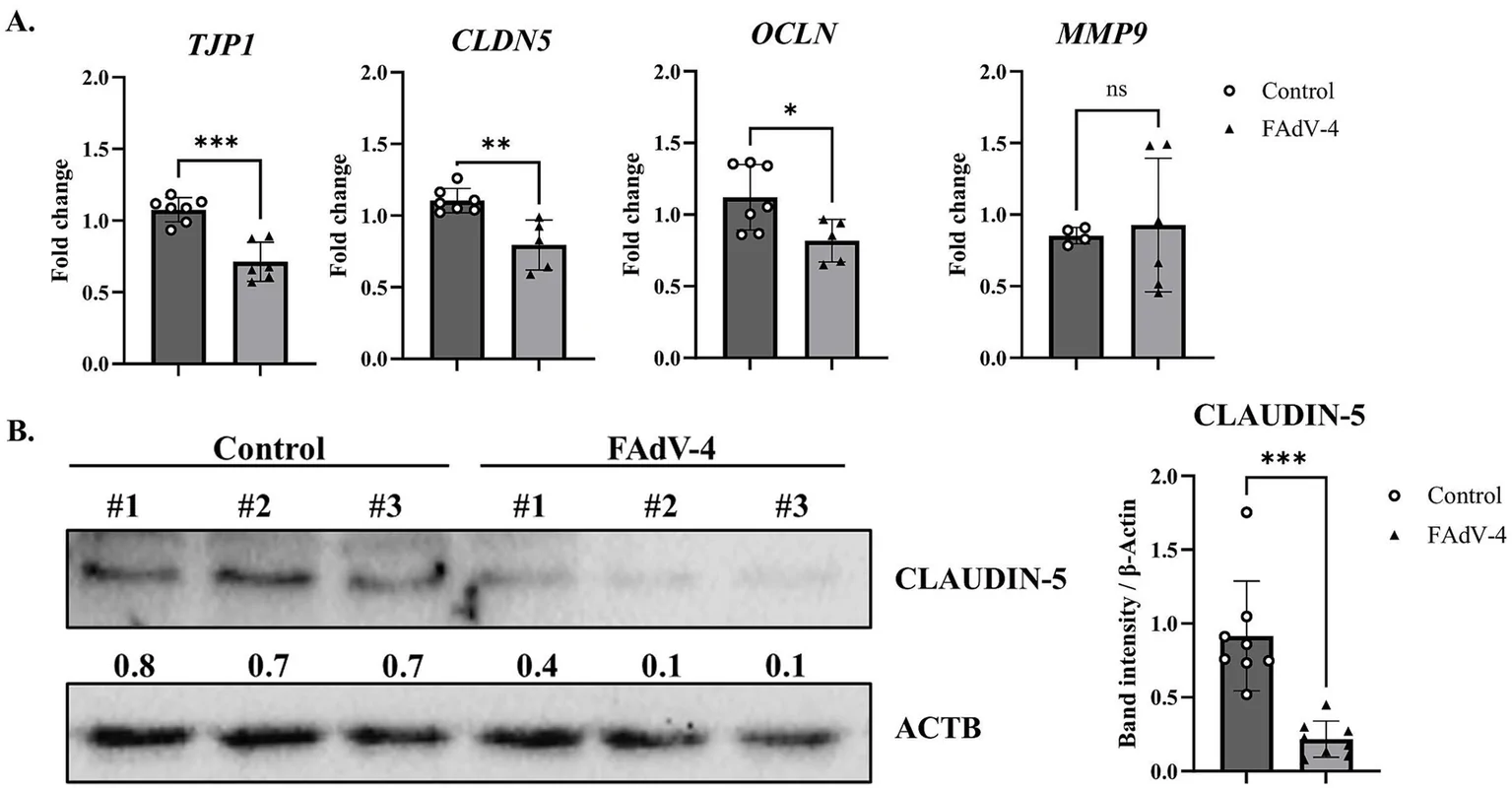
Changes in brain permeability-related genes expression during FAdV-4 infection. **(A)** Transcriptional level of tight junction proteins encoding genes were assessed by RT-qPCR. Gene expression levels were normalized to *ACTB* and presented as fold changes. RT-qPCR was conducted in duplicate, and data from three independent experiments (*n* = 2–4 per group) are presented. cDNA and protein were prepared from homogenized brain tissue. **(B)** CLDN5 protein expression was assessed by Western blot analysis. Band intensities were quantified using ImageJ software, and protein levels were normalized to *ACTB*. * *p* < 0.05, ** *p* < 0.01, *** *p* < 0.001.

### 3.7 Detection of transient viral DNA in the brain during FAdV-4 infection

Following the characterized neuroimmune response, we analyzed the presence of viral DNA in the brain and assessed its gross pathology. Quantitative analysis revealed that the relative CT values of the *hexon* gene were lower in the liver than in the brain (Supplementary Figure S5A), indicating a higher viral load in the hepatic tissue. In addition, the result of relative Ct value in the brain samples between 4 dpi and 5 dpi showed that the *hexon* DNA was higher in the 4 dpi brain samples (Supplementary Figure S5B). Qualitative examination by conventional PCR also observed the clear visualization of the *hexon* gene in each brain homogenate. A declining trend over time, where ± 94% of the brains of the FAdV-4 group at 4 dpi were positive for *hexon* DNA, whereas the detection rate dropped to approximately 86% at 5 dpi (Supplementary Figure S5C; Figure 7A). Moreover, the observation in gross pathology showed that the brain from the infected group was pale compared to the control group (Figure 7B). Our findings indicated a trend toward transient viral dissemination into the chicken brain during FAdV-4 infection.

**Figure 7:**
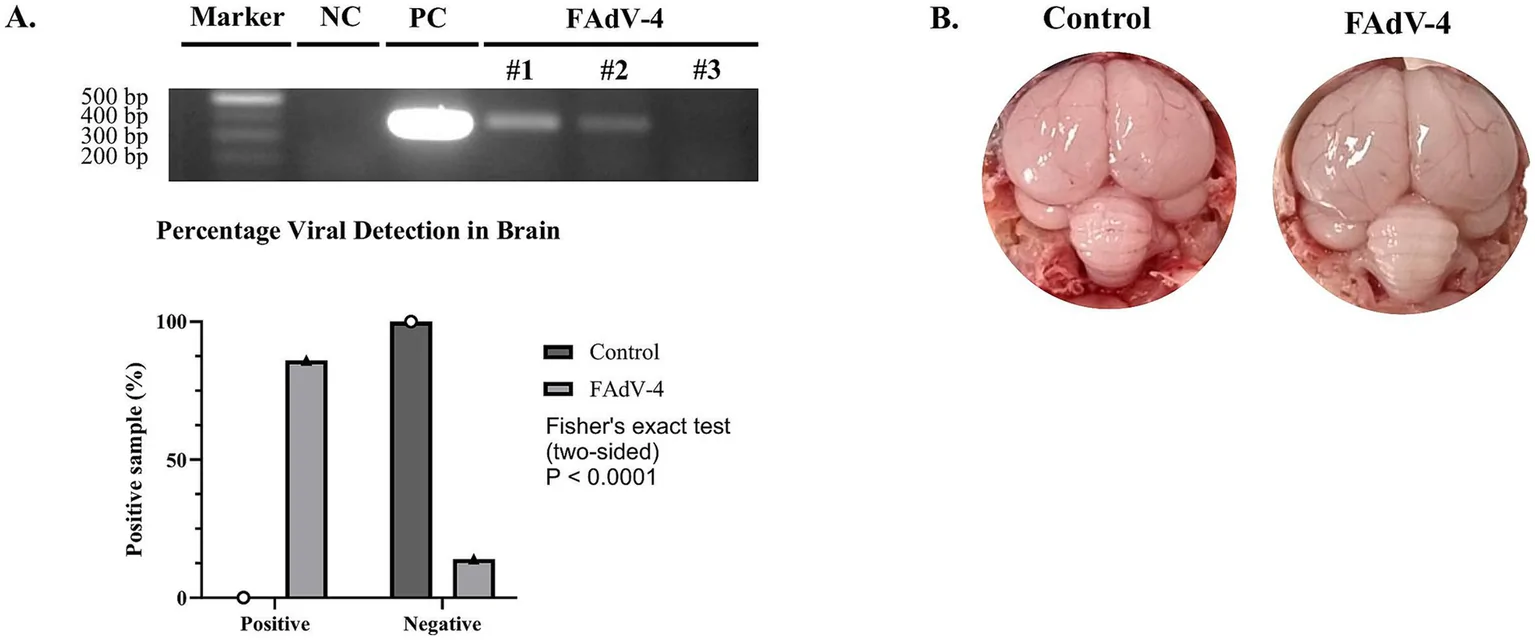
Viral detection in the brain and the brain’s gross pathology following FAdV-4 infection. **(A)** At 5 dpi, *hexon* DNA from FAdV-4 was detected in brain homogenate samples, a positive control obtained from isolated liver DNA. PCR amplification products from positive samples appear as a visible band on a 2% agarose gel. **(B)** The paler brain tissue is shown in the gross examination of an infected chicken group compared to the control group. The tissue pooled data are presented from three independent experiments (*n* = 2–4 chickens per group). NC, negative control; PC, positive control.

### 3.8 *In vitro* replication and infectivity of brain-derived viruses

To assess the replication capability of brain-derived viral particles, viral isolation was performed using supernatants obtained from brain homogenates. Supernatants from individual tissue samples that tested positive for FAdV-4 DNA were pooled and subsequently inoculated into LMH cells, a cell line commonly utilized for the propagation and vaccine production of avian adenovirus, including FAdV-4 (17). Throughout a 72-h observation period, cytopathic effects (CPE) characterized by cellular shrinkage, rounding, and detachment were observed across all virus-inoculated groups, whereas the negative control remained unaffected (Supplementary Figure S6). In the positive control group inoculated with liver-derived supernatants, typical CPE was fully observed at 12 h post-infection (hpi). In contrast, cells inoculated with brain-derived supernatants exhibited visible CPE starting >24 hpi, eventually reaching 100% CPE by 72 hpi. These findings demonstrated that FAdV-4 localized in the brain tissue retains its infectivity and viability, albeit requiring an extended incubation period to complete productive replication compared to liver-derived viruses.

## 4 Discussion

Following the FAdV-4 viral challenge, infected chickens exhibited clinical symptoms, including lethargy, weight loss, and sudden death starting at 3 dpi. Macroscopic pathological changes were characterized by hepatitis and splenomegaly. The splenomegaly observed concurrently with systemic immunosuppression characterized by the depletion of splenic immune cell populations, including activated macrophages, CD8 T cells, and γδ T cells. Beyond these clinical symptoms and lymphoid alterations, this study demonstrated that FAdV-4 may trigger a localized neuroimmune response within the chicken brain, a non-primary target organ. This central nervous system (CNS) organ involvement was characterized by the expansion of activated macrophages and cytotoxic CD8^+^ T cell subsets, alongside the increase of the pro-inflammatory cytokine *TNFA* mRNA. In addition to these neuroimmune alterations, we also observed the downregulation of TJ-related genes and the detection of FAdV-4 *hexon* DNA within the brain homogenate tissue from the infected group. Furthermore, the inoculation of LMH cells with pooled brain supernatants resulted in delayed CPE, confirming that the viral dissemination within the brain remains infectious and replication-competent. Collectively, our findings provide a comprehensive profiling of FAdV-4 pathogenesis in extrahepatic tissues, such as the brain.

FAdV-4 pathogenesis is widely documented in the liver, circulating blood, and lymphoid organs. These immune responses following the pathogenic changes, such as the increased frequencies of myeloid and lymphoid cells in the liver and circulating blood during viral challenge (7, 9). Systemic FAdV-4 dissemination consistently extended to various tissues, including lymphoid organs, where immune suppression occurs alongside lymphoid cell depletion (7, 33). This systemic depletion occurred as a result of structural and functional alterations within the spleen, such as splenic necrosis (33, 34). Consistent with these, the infected chickens in the present study exhibited pronounced hepatitis and altered splenic immune profiles following FAdV-4 challenge, evidenced by a depletion of splenic MHCII-expressing monocytes/macrophages, CD44^+^CD8^+^, CD44^+^γδ T, and CD8^+^γδ T cells. Together with the viral detection in the spleen, we also observed an increased spleen-to-body weight ratio in the infected chickens, indicating that the systemic alteration during acute FAdV-4 also occurred in our experimental model.

Beyond the lymphoid and hepatic alterations, extended FAdV-4 dissemination into the brain was observed (6). Microscopic changes have previously been observed in the brains of FAdV-4 infected chickens, including mild neuronal phagocytosis, vascular congestion, and perivascular edema, suggesting that the brain may also undergo inflammatory alterations during acute infection (11, 29). In this present study, monocytes/macrophages appeared morphologically enlarged, although their abundance was not quantitatively different by histological examination. Previous studies have shown that several avian viruses, including adenovirus (OTE strain), can induce characteristic cytopathic or morphological changes in chicken macrophages, with cell enlargement being associated with macrophage activation (35). In addition, hypervirulent FAdV-4 has also been reported to activate NLRP3 inflammation to induce IL-1β secretion in HD11 chicken macrophages (36), suggesting that FAdV-4 exposure can alter the functional activation state of macrophages.

MHCII expression reflects the antigen-presenting phenotype of activated myeloid cells (23). Consistent with this, our previous publication showed that virulent FAdV-4 infection has increased MHCII-expressing myeloid cells in the liver, accompanied by increased expression of myeloid-associated inflammatory genes, including IL-1β (7). In the brain, we observed a significant increase in macrophages and cytotoxic CD8^+^ T cell subsets in the sectioned brain tissue. Further immunophenotyping via flow cytometry revealed that the population of activated (MHCII^+^KUL01^+^), cytotoxic CD8^+^ T cells, CD8^+^γδ T cells, and activated γδ T cells in the brain was expanded. Taken together, these findings suggest that acute FAdV-4 infection is accompanied not only by morphological alterations of brain macrophages but also a shift toward an activated myeloid phenotype.

Due to the limited commercial products of avian-specific antibodies, the present flow cytometry analysis could not definitively distinguish whether this neuroimmune cell accumulation resulted strictly from the proliferation of tissue-resident immune cells or via the active infiltration of peripheral leukocytes. To supplement these cellular findings, the transcriptional profiles of microglia homeostatic genes, specifically *TMEM119*, *P2RY12*, and *CX3CR1,* were evaluated, revealing a statistically non-significant reduction in their mRNA expression levels within the brains of the infected group compared to the control group. The downregulation of microglial homeostatic markers is consistently associated with the functional transition of microglia from a resting, surveillance state toward an activated phenotype, upregulating pathways dedicated to phagocytosis and immune response (23, 37). Given that this microglial transcriptional decline was relatively subtle, the observed increased MHCII-expressing myeloid cells in the brain suggest a predominance of recruited circulating monocytes/macrophages.

The elevated MHC II expression by macrophages indicates viral antigen presentation and the initiation of adaptive immune responses (23, 38). Activated microglia/macrophage present antigens to T cells during neuroinflammation, leading to the infiltration of CD45^+^ immune cells at the sites of inflammation (15, 23). Among these lymphocyte populations, recruited CD8^+^ cytotoxic T cells undergo clonal expansion to eliminate infected cells (15, 39). These effector cells recognize foreign antigens presented by MHC I molecules and mediate targeted cytolysis of virus-infected cells (40, 41). FAdV-4 infection led to an increased number of activated CD8^+^ T cells in the brain, reflecting an active adaptive immune response in this study. Cytotoxic activity, specifically mediated by γδ T cells offers a more rapid immune response due to their capacity to recognize antigens in an MHC-independent manner, thereby conferring a high cytolytic potential (25). These γδ T cells activation pathway, including the recognition of stress-induced antigens, phosphoantigens, and other non-peptide molecules (42, 43). In this present study, a significantly higher frequency of activated γδ T cells and γδ CD8^+^ T cells was quantified within the brain at 5 dpi. These findings demonstrate that acute FAdV-4 infection triggers the expansion of cytotoxic T cell subsets and an activated CD8^+^ T cell population in the brain. However, the precise functional and cytolytic efficacy of these specific lymphocyte populations remains to be definitively elucidated in future studies.

Lymphocyte activation, including T cells, involves activation of nuclear factor-kappa B (NF-κB), an important transcription factor that regulates immune responses and inflammatory gene expression during infection and cancer. The signaling pathway to activate NF-κB is divided into canonical, activated by antigens, CD28, 4-IBB, GITR co-stimulators, TNF, and IL-1, and the non-canonical pathway, which is triggered by CD40, BAFF-R, and LTβ-R (44). Specifically, the canonical activation of NF-kB in T-cell receptor (TCR), which recognizes antigen presentation by MHC molecules, relies on caspase recruitment domain-containing protein 11 (CARD11) to assemble the CARMA-BCL10-MALT1 (CBM) complex before triggering downstream TAK1 and IKK complexes (44, 45). The non-significant changes in mRNA CARD11 expression were observed in our study. These suggest that the TCR canonical pathway did not dominate the mechanism for activating CD8^+^ and γδ T cells in our model. This transcriptional pattern aligns with a previous non-neurotropic avian viral infection model, including FAdV-4, which documented a lack of CARD11 expression within the brain (12). Besides the minimal involvement of CARD11, the evaluation of mRNA expression levels for key chemokine ligands and receptors, specifically *CCR5, CCL5,* and *CXCL13*, also remained unchanged in the brain tissue of the infected group. In contrast, in the inflamed brain tissue caused by neurotropic viral infection, such as Tembusu virus (TMUV), or other inflammatory diseases, increased *CCL5* expression actively attracts *CCR5*-expressing cells (13), while macrophages express *CXCL13* to recruit B cells to infiltrate the brain (46). Collectively, our results suggest that localized parenchymal chemokines may not actively regulate peripheral immune cell infiltration and that alternative signaling routes are likely responsible for activating these T-cell subsets.

Alternatively, NF-κB activation can be directly engaged via TNF receptors (TNFR), a receptor for TNF-α cytokines. This pathway and the canonical TCR-CBM cascade ultimately converge at the same downstream IKK complex to trigger NF-κB nuclear translocation (44). We observed that the mRNA expression of *TNFA* was significantly higher in the infected group than in the control group. This may indicate that the upregulation of the *TNFA* gene expression may also play a role in activating T cells. *TNFA* is also a potent pro-inflammatory cytokine with cytotoxic effects, primarily produced by activated T cells, including CD8^+^ T cells (Tc1) and macrophages (47, 48). Together, these findings suggest that besides inducing inflammation in the brain, the increased *TNFA* gene expression potentially contributes to the bystander activation of T cells in the brain during acute FAdV-4 infection.

The influx and subsequent bystander activation of peripheral immune cells depend heavily on the structural permeability of the blood–brain barrier (BBB). The inflammatory milieu within the CNS is known to affect TJ protein expression in brain endothelial cells, a process often mediated by matrix metalloproteinases (MMPs) that degrade TJ structural elements, such as *ZO1* and *OCLN*, during avian neurotropic viral infections (13, 14, 32). In addition, overexpression of pro-inflammatory markers in the periphery can also impair TJ function. For example, persistent systemic inflammation in LPS-induced models converts microglia into a phagocytic state, leading to a subsequent reduction in claudin-5 (CLDN5) expression within murine brains (16). In the brain tissues of our infected chickens, we found that claudin-5 protein expression was reduced, and the transcript levels of TJ-related genes (*CLDN5, TJP1, and OCLN*) were significantly decreased. These changes suggest that the loosened cellular junctions likely facilitate the passive dissemination of circulating peripheral immune cells, including myeloid and cytotoxic lymphocytes, and viral particles into the brain. However, further research involving direct BBB permeability assessment should be conducted.

The extended FAdV-4 dissemination into the brain is detectable during disease progression, although the relative CT value of *hexon* DNA in the brain was lower than that quantified within hepatic tissue. Once FAdV-4 establishes its presence within the cerebral microenvironment, it likely triggers an immediate interaction with the localized host innate defense. Type I IFNs, including *IFNA* and *IFNB*, are major antiviral cytokines produced by innate immune cells during viral infections and play a critical role in controlling viral replication (40). Additionally, beyond antiviral functions, type I IFNs contribute to the maintenance of BBB endothelium integrity by counteracting TNF-α-mediated barrier disruption (49). In our model, while host *IFNA* mRNA expression levels remained relatively stable, the transcriptional expression of *IFNB* was significantly suppressed within the infected brain tissues. Previously, FAdV-4 has been reported to suppress IFNB signaling through protein kinase R (PKR) degradation and viral non-structural ORF1B proteins targeting IRF7 in cell lines (50, 51). This selective downregulation suggests that the localized host type I IFN response is partially compromised in the brain, which potentially contributes to both permissible viral propagation and compromised endothelial TJ. However, the specific cellular mechanisms underlying this suppressed *IFNB* profile require direct functional investigation in future studies.

Given that in this study we observed the transient FAdV-4 viral dissemination in the brain, to definitively validate whether the cerebral FAdV-4 retained functional replication-competency under this suppressed *IFNB* milieu, optimized brain-derived supernatants from the infected chickens were inoculated onto LMH cell cultures. The brain-derived supernatants successfully induced observable cytopathic effects (CPE) *in vitro*, confirming the existence of viable viral particles in the brain tissue. However, the onset of these cytopathic changes was delayed compared to the LMH cells inoculated with liver-derived supernatant. This onset of CPE differences aligns with the lower viral detection in the brain homogenate samples. As a primary target organ, FAdV-4 highly replicates in the liver, thus causing more rapid and pronounced cytopathic changes, including loss of adherence, cell shrinkage, rounding, and chromatin condensation (19, 52).

### 4.1 Limitations of the study

Our present study suggested that FAdV-4 challenge potentially triggered the downregulation of TJ-related genes and protein expression, thus increasing the brain immune cell population. However, direct *in vivo* barrier permeability assays were not performed. Future studies utilizing specific endothelial tracer kinetics are warranted to definitively map the physical permeability alterations of the CNS endothelium during FAdV-4 infection. Another limitation of this study is that the transcriptional analysis was restricted to a selected panel of immune- and tight-junction-related genes. Therefore, future transcriptomic analysis, such as involving the RNA-seq method, will be necessary to identify additional pathways that may be involved in neuroimmune and barrier-associated responses following acute FAdV-4 infection.

## 5 Conclusion

In conclusion, we demonstrated alterations in the brain immune cell composition during acute FAdV-4 infection, which was characterized by an increased in MHCII-expressing monocytes/macrophages, activated cytotoxic T cells, activated γδ T cells, and cytotoxic γδ T cells population, together with elevated *TNFA* mRNA expression. These immunological alterations were accompanied by a decreased expression of TJ-related genes, particularly *CLDN-5,* as well as reduced CLDN-5 protein expression, indicating concurrent immune and barrier-associated alterations in the brain. However, whether the expanded brain immune cell populations originate from activation or proliferation of tissue-resident cells, recruitment of circulating peripheral leukocytes, or both remains to be determined. Furthermore, infectious FAdV-4 was recovered from brain homogenates, supporting the involvement of the brain during acute FAdV-4 infection. Overall, this study provides new insights into the immune and barrier-associated changes occurring in a non-primary target organ of FAdV-4 infection.

## Data Availability

The original contributions presented in the study are included in the article/Supplementary material, further inquiries can be directed to the corresponding author.
