# Supplementary material for: Neuroimmune responses in the chicken brain during acute fowl adenovirus serotype 4 infection
Source: Front Vet Sci. 2026 Sep 23;13:1964202. doi: 10.3389/fvets.2026.1964202 (PMC13643516; doi:10.3389/fvets.2026.1964202)
Supplement: Supplementary file 1 [file Data_Sheet_1.pdf]

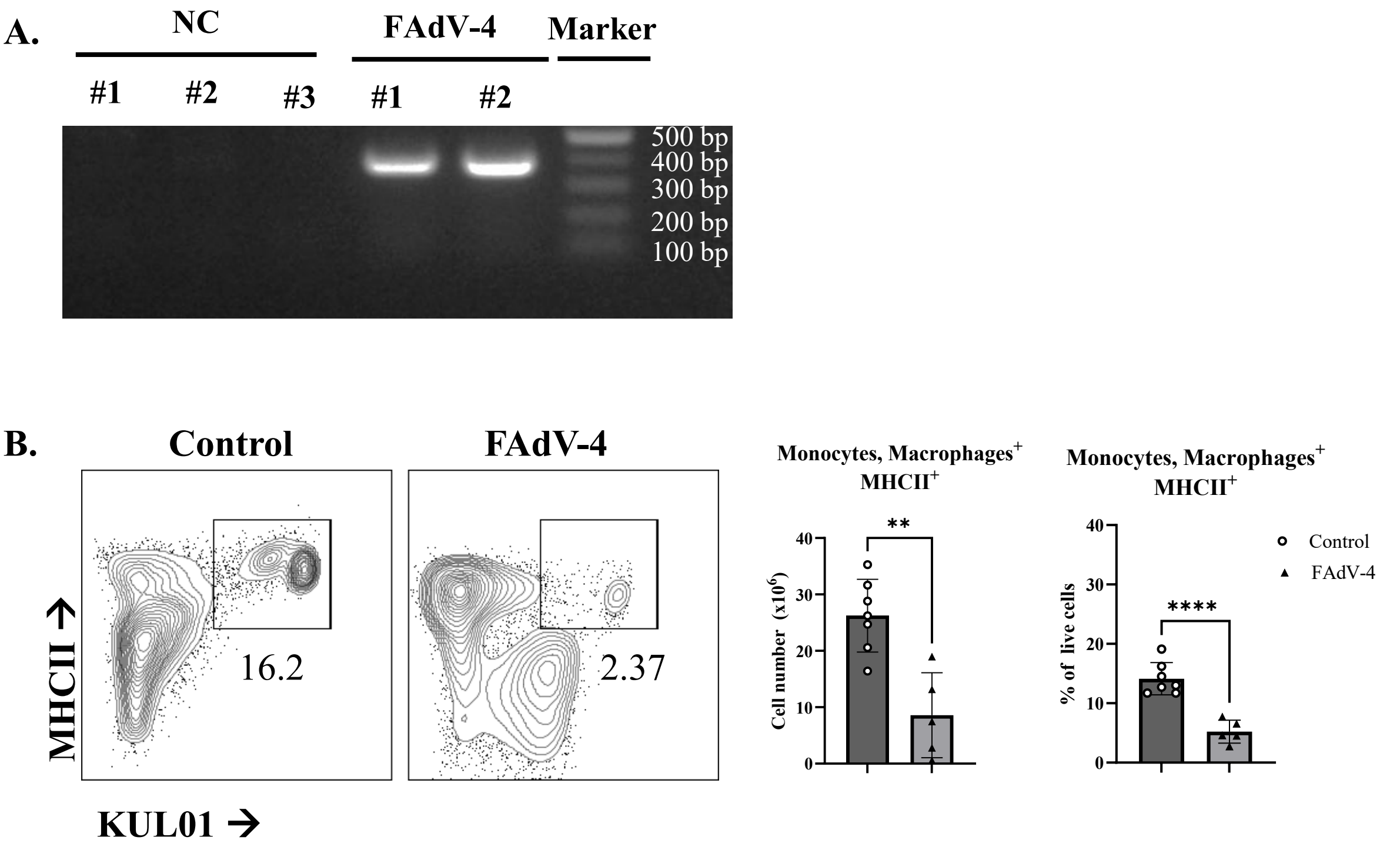

Supplementary Figure S1. (A) *Hexon* DNA of FAdV-4 detection in the spleen. NC = negative control. **(B)** Alterations in splenic monocyte/macrophage populations following FAdV-4 infection. Cell numbers and the frequencies of MHC II–expressing monocytes/macrophages in the spleen were analyzed by flow cytometry at 5 dpi. Splenocyte data were collected from 2–4 chickens per group and a representative plot is shown. *p<0.05, **p<0.01, ***p<0.001, ****p<0.0001.

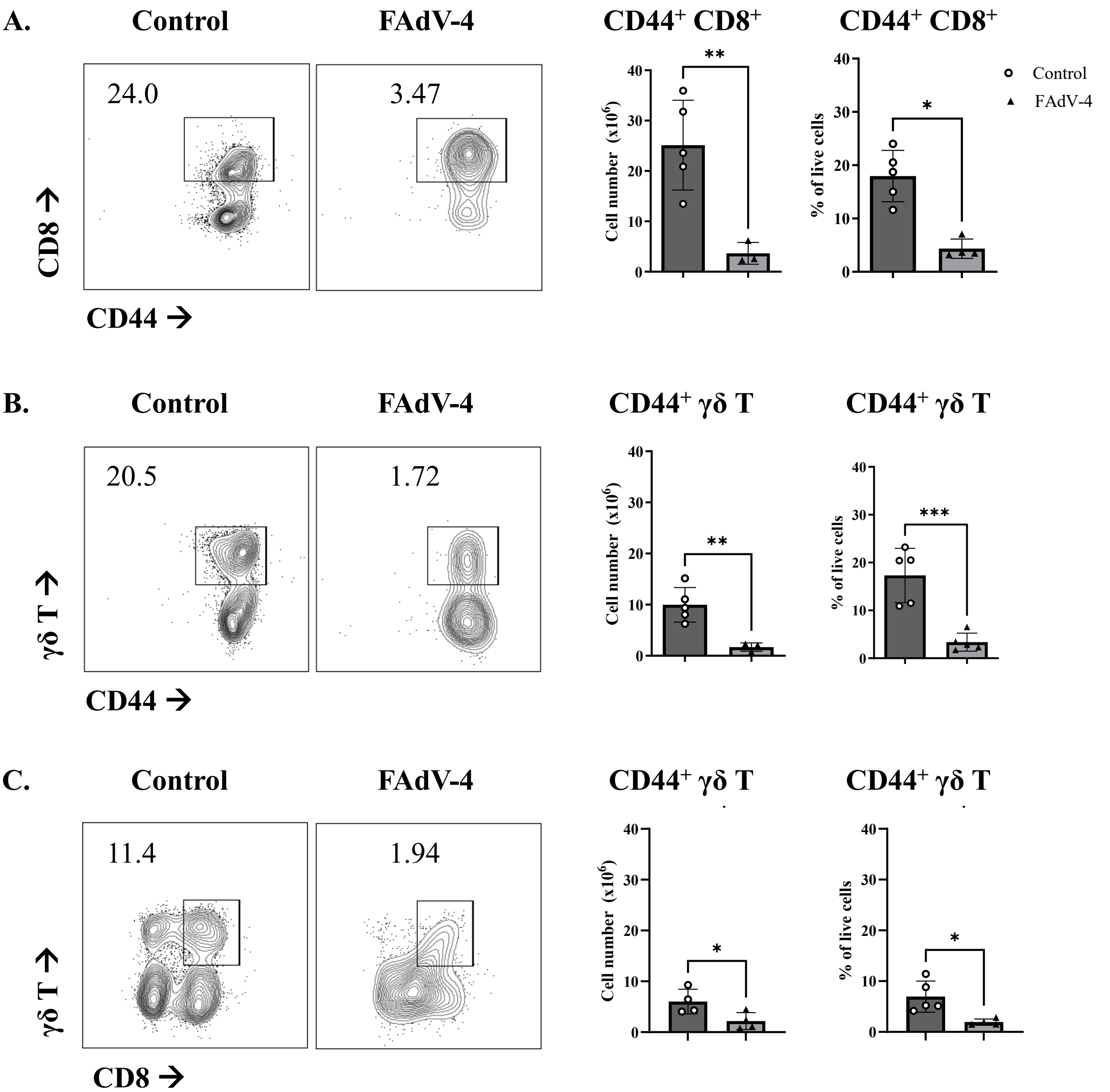

Supplementary Figure S2. Changes in T cell subsets in the spleen following acute FAdV-4 infection. The cell numbers and frequencies of **(A)** activated T cytotoxic cells ($CD8^+CD44^+$), **(B)** activated γδ T cells ($CD44^+$ $γδ^+$) and **(C)** γδ $CD8^+$ T cells are presented. Splenocytes data were collected from 2 to 4 chickens per group, and a representative plot is shown. *$p<0.05$, **$p<0.01$, ***$p<0.001$, ****$p<0.0001$.

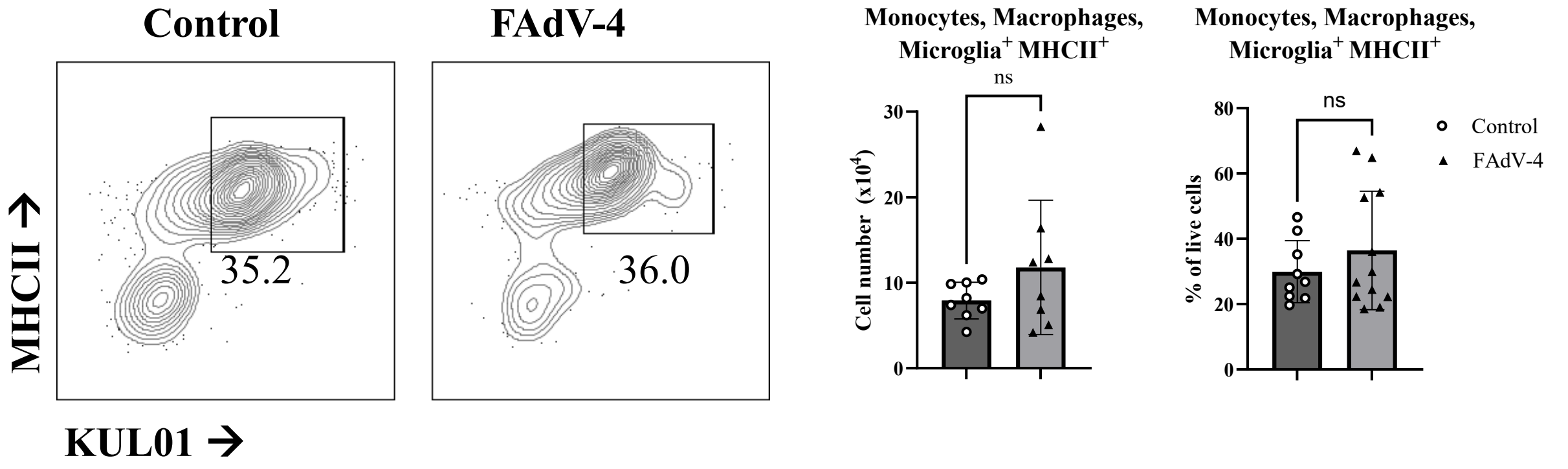

Supplementary Figure S3. Flow cytometric analysis of MHC II^{+} myeloid populations (monocytes, macrophages, and microglia) in the brain at 4 dpi. Frequency and total cell numbers were determined from brain homogenates (n = 2–4 chickens per group). The data shown is polled from three independent experiments.

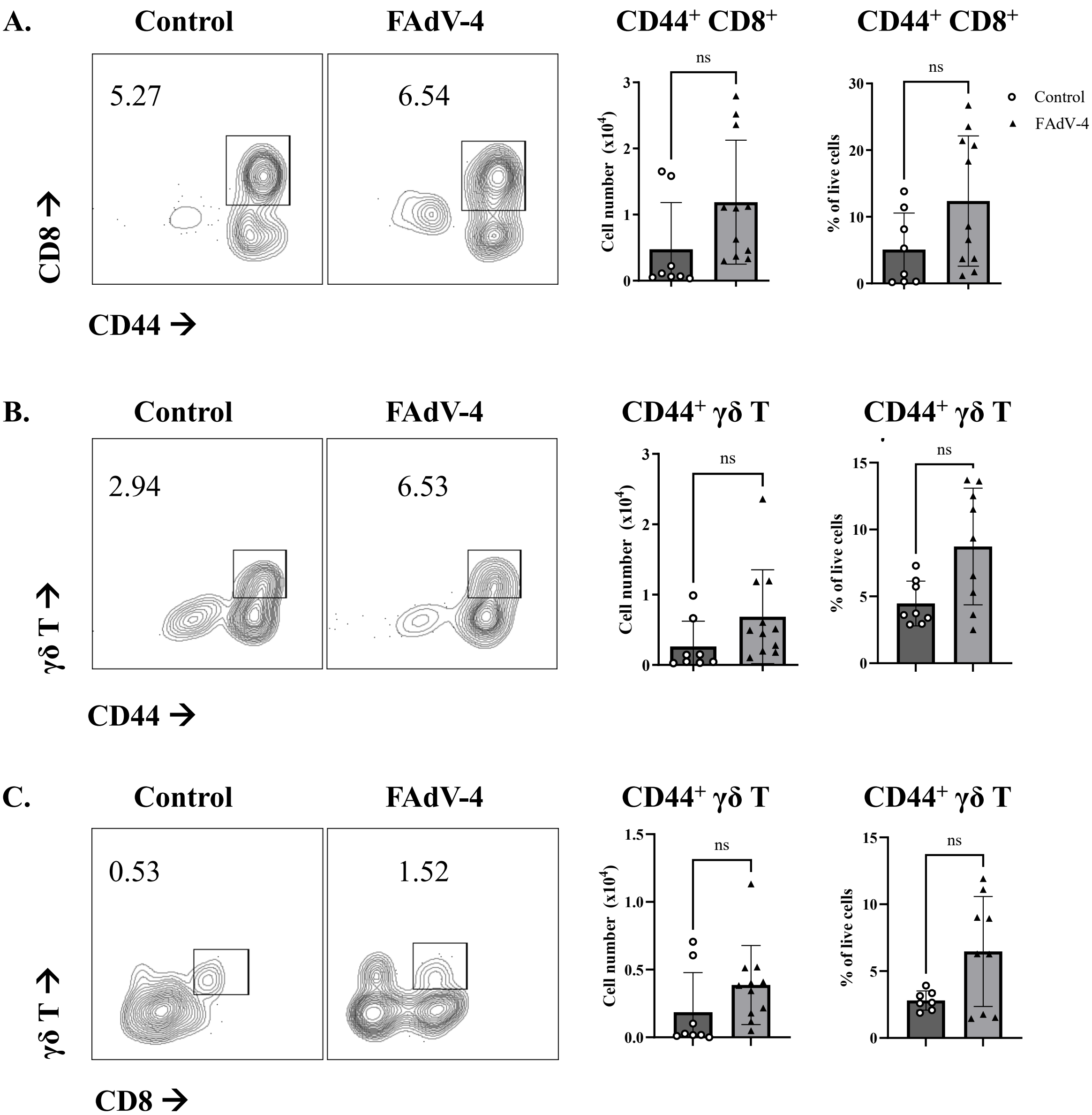

Supplementary Figure S4. Assessment of brain T cell activation post-FAdV-4 infection. Flow cytometric data show the distribution and abundance of activated **(A)** CD8$^+$, **(B)** CD44$^+$ γδ T, and **(C)** CD8$^+$ γδ T cell subsets from brain tissue at 4 dpi. All results are derived from three separate experiments, using 2 to 4 chickens per group. Significance levels: $p < 0.05$ (*); p < 0.01 (**).

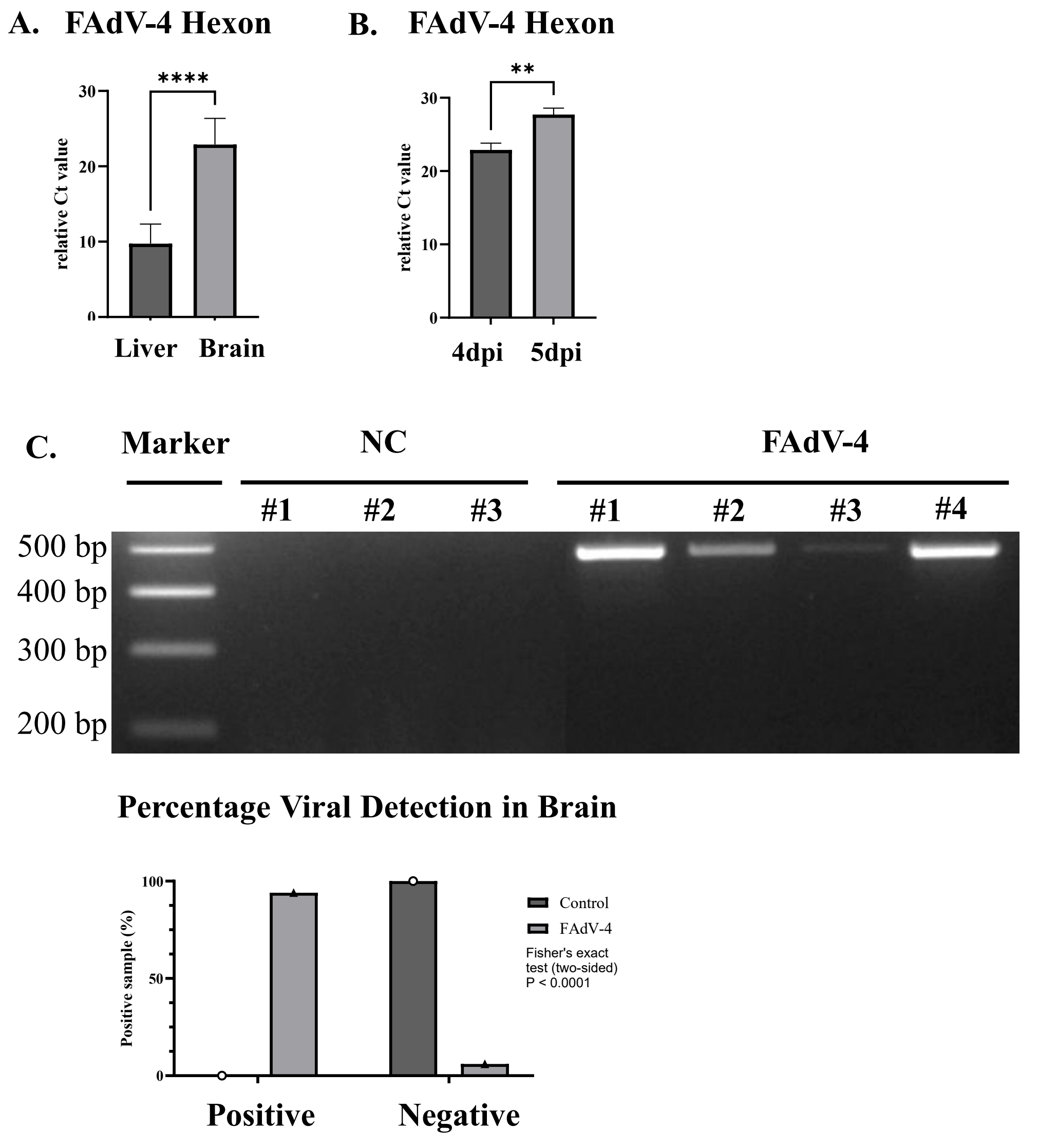

Supplementary Figure S5. **(A)** The relative levels of viral DNA were assessed by RT-qPCR, run in duplicate, and the results were expressed as Cycle Threshold (Ct) values. At 4 days post-infection (dpi), Ct values in the brain were notably higher compared to those in the liver, indicating a lower viral DNA presence in the brain tissue. **(B)** Comparison of relative Ct values of Hexon in the brain between 4 dpi and 5 dpi. Viral DNA remained detectable in the brain at 5 dpi. **(C)** Viral detection by conventional PCR in the brain samples at 4 dpi. The graph indicated the percentage (%) of positive and negative from the pooled tested samples. Data are presented as mean ± SD (n = 2–4 per group).

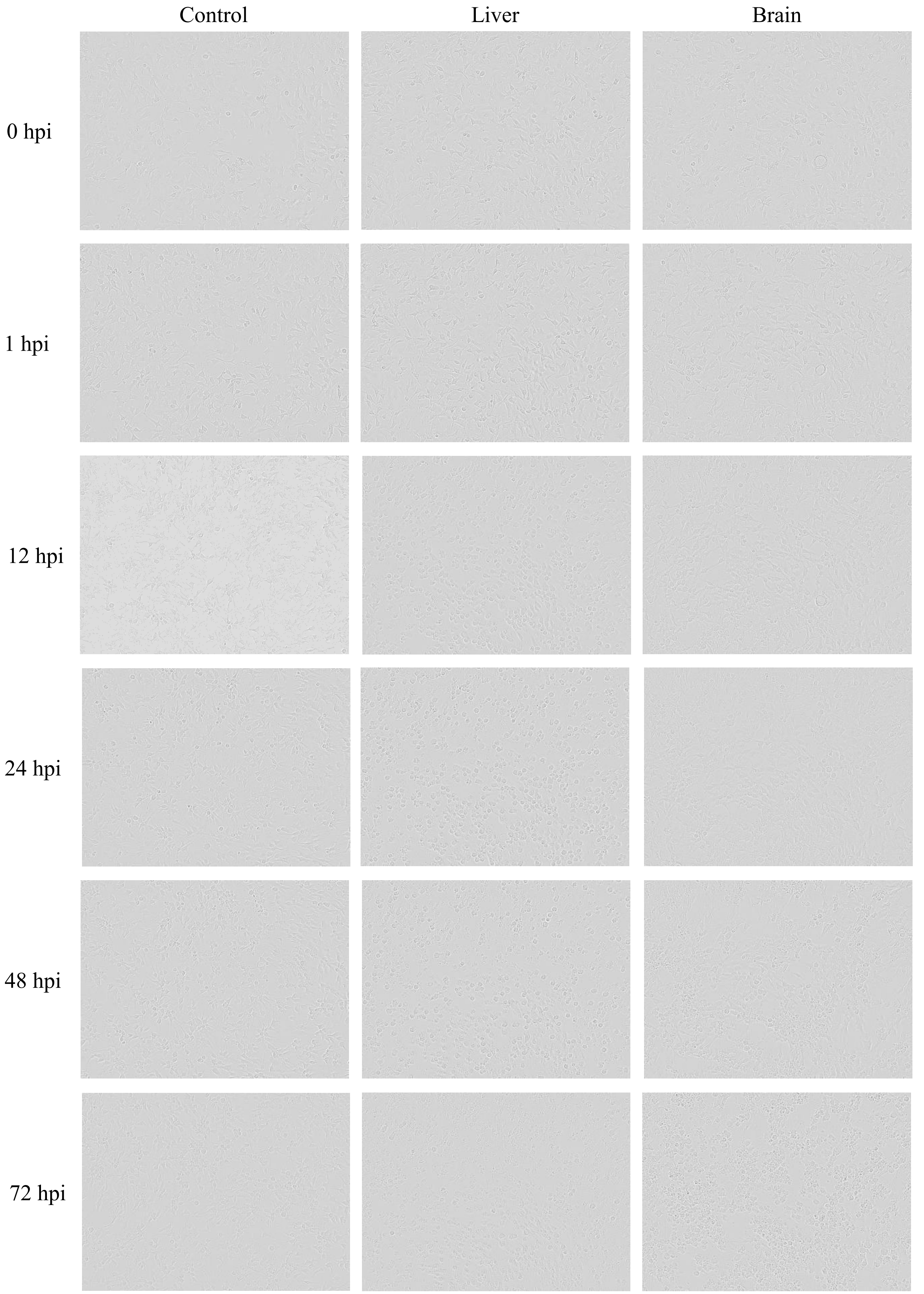

Supplementary Figure S6. The result of LMH cells inoculated with tissue-derived supernatant treatment. The CPE was observed until 72 hpi at x20 magnification.
